# Genes affected by mouse mammary tumor virus (MMTV) proviral insertions in mouse mammary tumors are deregulated or mutated in primary human mammary tumors

**DOI:** 10.18632/oncotarget.682

**Published:** 2012-10-18

**Authors:** Robert Callahan, Uma Mudunuri, Sharon Bargo, Ahmed Raafat, David McCurdy, Corinne Boulanger, William Lowther, Robert Stephens, Brian T. Luke, Claudia Stewart, Xiaolin Wu, David Munroe, Gilbert H. Smith

**Affiliations:** ^1^ Cell and Cancer Biology Branch, National Cancer Institute; Bethesda, MD, USA; ^2^ Advanced Technology Program, SAIC-Frederick, Inc., NCI-Frederick, Frederick, MD, USA; ^3^ Laboratory of Molecular Technology, SAIC-Frederick, Inc., NCI-Frederick, Frederick, MD, USA

**Keywords:** mouse mammary tumor virus, premalignant lesions, mammary tumors, genes, human breast carcinomas, metastases

## Abstract

The accumulation of mutations is a contributing factor in the initiation of premalignant mammary lesions and their progression to malignancy and metastasis. We have used a mouse model in which the carcinogen is the mouse mammary tumor virus (MMTV) which induces clonal premalignant mammary lesions and malignant mammary tumors by insertional mutagenesis. Identification of the genes and signaling pathways affected in MMTV-induced mouse mammary lesions provides a rationale for determining whether genetic alteration of the human orthologues of these genes/pathways may contribute to human breast carcinogenesis. A high-throughput platform for inverse PCR to identify MMTV-host junction fragments and their nucleotide sequences in a large panel of MMTV-induced lesions was developed. Validation of the genes affected by MMTV-insertion was carried out by microarray analysis. Common integration site (CIS) means that the gene was altered by an MMTV proviral insertion in at least two independent lesions arising in different hosts. Three of the new genes identified as CIS for MMTV were assayed for their capability to confer on HC11 mouse mammary epithelial cells the ability for invasion, anchorage independent growth and tumor development in nude mice. Analysis of MMTV induced mammary premalignant hyperplastic outgrowth (HOG) lines and mammary tumors led to the identification of CIS restricted to 35 loci. Within these loci members of the *Wnt*, *Fgf* and *Rspo* gene families plus two linked genes (*Npm3* and *Ddn*) were frequently activated in tumors induced by MMTV. A second group of 15 CIS occur at a low frequency (2-5 observations) in mammary HOGs or tumors. In this latter group the expression of either *Phf19* or *Sdc2* was shown to increase HC11 cells invasion capability. *Foxl1* expression conferred on HC11 cells the capability for anchorage-independent colony formation in soft agar and tumor development in nude mice. The published transcriptome and nucleotide sequence analysis of gene expression in primary human breast tumors was interrogated. Twenty of the human orthologues of MMTV CIS associated genes are deregulated and/or mutated in human breast tumors.

## INTRODUCTION

Mutations contribute to the evolution of normal mammary epithelium through premalignant lesions to malignancy and metastases [1]. Our approach was to use the mouse mammary tumor virus (MMTV) induced mammary tumor model to identify genetic pathways altered by insertional mutation in multiple lesions that contribute to mouse mammary tumorigenesis by expansion of the mutated cell (reviewed in [2]). The rationale is that mutations in similar pathways may also contribute to human breast carcinogenesis.

Different strains of MMTV have been distinguished based on the types of preneoplastic mammary lesions and mammary tumors they induce in the mouse [3]. Thus MMTV from inbred C3H or feral *Mus musculus musculus* CzechII (designated CZ) mice induces transplantable preneoplastic hyperplastic alveolar nodules (HANs) that can be maintained indefinitely as mammary premalignant hyperplastic outgrowth (HOGs) lines (reviewed in [2]). Within these populations pregnancy independent mammary tumors develop stochastically. In contrast, MMTV from inbred BR6, GR or feral *Mus spretus* (designated SP) mice develop pregnancy dependent mammary “plaques” that have been described as “a system of branching tubules often with bulbous ends” [3-7].

C3H/He and BR6 inbred mouse strains with high mammary tumor incidence were used to identify the initial common integration sites (CIS) for MMTV in mammary tumor DNA [8]. Czech II mice have not been selected for a high incidence of mammary tumors and lack endogenous MMTV genomes [9]. We have collected over 40 HOG lines from CzechII mice and both mammary tumors and lung metastases that developed within these premalignant lesions. Southern blot analysis confirmed the clonal nature of progression in this model [3].

Previous screens of MMTV^C3H^ induced mammary tumors in C3H or Balb/c mice that had been foster nursed on C3H mice (designated Balb/cfC3H) for MMTV CIS in mammary tumors have been reported [10-12]. In the present study we have compared the CIS in mammary tumors for two other strains of MMTV (MMTV^CZ^ and MMTV^SP^) that had been foster nursed on to Balb/c mice (Balb/cf MMTV^CZ^ and Balb/c MMTV^SP^, respectively) with those for MMTV^CZ^ in CzechII mice. In addition we have compared the MMTV CIS for MMTV^CZ^ in mammary preneoplastic HOGs, HOG-derived mammary tumors and lung metastases. An inverse PCR protocol was adapted to a high-throughput platform to identify MMTV-host junction fragments and to determine their nucleotide sequences [13, 14] in a large panel of MMTV-induced lesions. The published transcriptome and nucleotide sequence analysis of primary human breast tumors was interrogated to ascertain whether the expression of the human orthologues of these new MMTV CIS target genes was deregulated or mutated in human breast cancer. Three newly discovered CIS genes were assayed for their capability to confer on HC11 mouse mammary epithelial cells the ability for invasion, anchorage independent colony formation in soft agar and/or tumorigenesis in athymic nude mice.

## RESULTS

### High throughput identification of MMTV integration sites in mouse mammary tumors

Premalignant mammary hyperplastic outgrowth lines (HOGs) were derived from hyperplastic alveolar nodules (HANs). HOGs only grow in the mammary fat pad and never exceed the limits of the fat pad. These populations will not overgrow normal mammary gland in the same fat pad and are not transplantable in an ectopic site [8]. HOGs, mammary tumors and metastases were collected from and/or maintained in CzechII feral mice. MMTV^CZ^ and MMTV^SP^ viruses were transmitted by foster nursing to Balb/c mice (Balb/cf MMTV^CZ^ and Balb/cf MMTV^SP^), where they induced, respectively, pregnancy independent and pregnancy dependent tumors that progressed to pregnancy-independence after a few parities (see Materials and Methods). Our primary tumor panel consisted of the premalignant HOGs and mammary tumors from Czechll feral mice and Balb/cf MMTV^CZ^ and Balb/cf MMTV^SP^ mammary tumors.

The inverse PCR [13, 14] approach to cloning host-viral junction fragments (designated retroviral integration site, RIS) was adapted to a high throughput platform (see Materials and Methods). Of 642 host-viral junction fragments, 591 mapped to unambiguous genomic locations, while the remaining fragments mapped to repetitive locations that could not be resolved. Using the statistical analysis procedure described by Mikkers et. al. [15], the parameters for defining clusters of MMTV RIS as candidate common integration site (MMTV CIS) was determined (see Materials and Methods and Table1). The effect of MMTV integration on the expression of genes was demonstrated by microarray analysis (MA#1 and MA#2, Tables 2 and 3) using a new visualization tool (Materials and Methods) which can be viewed on line at: [16] and/or by reverse transcriptase polymerase chain reaction (RT-PCR, Tables 2 and 3) analysis (Supplemental Figures 1 and 2). A final MMTV CIS gene list was then compiled based on the combined results of these steps and is presented in Tables 2 and 3.

**Table 1:** Frequency of RIS and CIS cluster distance in Mammary HOGs and Mammary tumors

	HOGs & HOG deriv. Tumors	CzechII	Balb/cf MMTV(CZ)	Balb/cf MMTV(SP)
# of Tumors	127	123	54	85
# of RIS	89	190	150	213
				
2 cluster distance	305277	46206	46206	46206
				
3 cluster distance	4317269	653464	653464	653464

The numbers of HOGs and mammary tumors as well as the number of independent RIS per tumor panel is indicated. Identification of CIS or clusters of RIS near a gene was accomplished using the statistical analysis procedure describe by Mikkers et al. [15]. Calculation of CIS clusters was done at the website: [44]. The distance within which 2 or 3 MMTV insertions have to occur for a total of 591 insertions (CzechII, Balb/cfMMTV(CZ) and Balb/cfMMTV(SP)), with a probability of < 0.01 is indicated. This approach led to the identification of 6 CIS and proximal genes in HOGs and HOG derived CzechII mammary tumors; 18 CIS and proximal genes in spontaneous CzechII mammary tumors; 16 CIS and proximal genes in spontaneous Balb/cfMMTV(CZ) mammary tumors and 20 CIS and proximal genes in spontaneous Balb/cfMMTV(SP) mammary tumors (see also Tables 2 and 3). Distance numbers = base pairs.

**Table 2:** Common Integration Sites for Feral strains of MMTV in Mouse Mammary Tumors^1^

Gene	Chr.	#insert. Events	CzechII HOGs	HOG Tumors	CzechII	Balb/cf MMTV(CZ)	Balb/cf MMTV(SP)	MA #1	MA #2	RT-PCR
Phf19	2	3	0	0	2	1	0	N	N	Y
Sfmbt2	2	2	0	0	1	0	1	NT	Y	Y
Fgr	4	2	0	0	0	0	2	NT	Y	Y
Gm12690	4							NT	NT	NT
Gm3917	4	3	0	0	2	1	0	NT	NT	NT
Gm12684	4							NT	NT	NT
Pdgfra	5	3	1	2	0	0	0	Y	NT	Y
Fezf1	6	3	0	0	2	1	0	Y	Y	Y
Tacstd2	6	2	0	0	0	1	1	NT	Y	Y
Fgf15	7							Y	Y	Y
Fgf3	7	17	0	1	7	2	7	Y	Y	Y
Fgf4	7							Y	Y	Y
Foxl1	8	2	0	0	0	2	0	NT	NT	Y
Gm6856	8	2	0	0	1	0	1	NT	NT	NT
Rspo3	10	4	0	0	1	1	2	NT	NT	Y
Gm7000	10	5	0	0	0	3	2	NT	NT	NT
Gm8613	10	2	0	0	0	1	1	NT	NT	NT
Gm2019	13	2	0	0	0	1	1	NT	NT	NT
Gm5770	14	2	0	0	0	1	1	NT	NT	NT
Wnt10b	15							Y	Y	Y
Wnt1	15	20		1	5	5	9	Y	Y	Y
Ddn	15							Y	Y	Y
Rspo2	15	22	10	1	5	1	5	Y	Y	Y
Sdc2	15	4	0	0	1	0	3	Y	Y	Y
Npm3	19							Y	NT	Y
Fgf8	19	5	0	0	3	1	1	Y	NT	Y
Col4a5	X	2	0	1	0	0	1	Y	NT	Y

1 The number of MMTV insertion events is indicated and in which tumor panels they occurred. Genes underlined comprise a cluster of genes that are affected by MMTV integration. In a cluster of genes the number of insertion events is given for only one gene in the cluster. MA#1 and MA#2 represent the results of two independent microarray analysis of mammary tumor RNAs. The results can be visualized at the website [16]. RT-PCR summarizes the results of gel analysis of RT-PCR products shown in Supplemental Figures 1 and 2. N= not up regulated with respect to mammary gland from 15 day pregnant mice, Y= yes up regulated with respect to mammary gland from 15 day pregnant mice, NT=not tested, Chr.= chromosome number

**Table 3:** Intragenic Common Integration Sites for MMTV in Mouse Mammary Tumors^1^

Gene	Chr.	#insert. Events	CzechII HOGs	HOG Tumors	CzechII	Balb/cf MMTV(CZ)	Balb/cf MMTV(SP)	MA #1	MA #2	RT-PCR
Nckap5	1	3	0	0	1	0	2	N	N	Y
Pde3a	6	2	0	0	1	0	1	NT	NT	Y
Usp31	7	2	0	0	2	0	0	N	N	Y
Nxn	11	2	0	0	1	1	0	NT	NT	Y
Gm4260	12	2	0	0	2	0	0	NT	NT	NT
Phactr1	13	2	0	0	0	0	2	NT	Y	N
Cadm2	16	2	0	0	1	0	1	NT	Y	Y
Kcnj6	16	2	0	0	2	0	0	N	N	N
Notch4	17	3	0	0	0	1	2	Y	NT	Y

**Figure 1:**
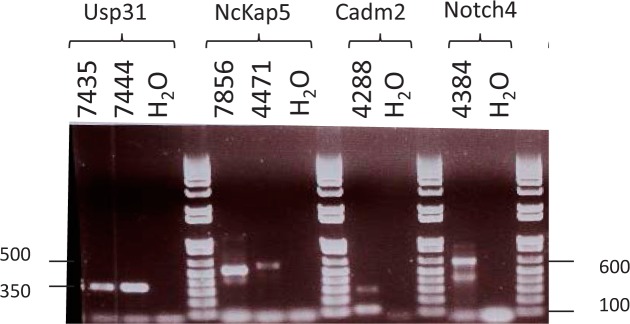
Agarose Gel Analysis of Intragenic RT PCR Products RT-PCR verification of MMTV CIS intragenic expression of MMTV LTR U5-host chimeric mRNA from MMTV induced mammary tumors. Agarose gel (2%) electrophoresis of the PCR product using nested MMTV LTR U5 and host exon primers (Supplementary Table 2). The size of the fragments, in base pairs (bp) is indicated on the Y-axis and is 350 bp for *Usp31*, 500 bp for *Nckap5*, 100 bp for *Cadm2* and 600 bp for *Notch4*. The name of the gene and a H_2_O control is indicated.

1 Intragenic MMTV CIS genes. The number of MMTV insertion events is indicated and in which tumor panels they occurred. MA#1 and MA#2 represent the results of two independent microarray analysis of mammary tumor RNAs. The result can be visualized at the website [16]. RT-PCR summarizes the results of gel analysis of RT-PCR products shown in Figures 1 and Supplemental Figure 1. N= not up regulated with respect to mammary gland from 15 day pregnant mice, Y= yes up regulated with respect to mammary gland from 15 day pregnant mice, NT=not tested, Chr.= chromosome number.

### MMTV CIS genes in Czechll, Balb/cf MMTV^CZ^ and Balb/cf MMTV^SP^ induced mammary tumors

The list of MMTV CIS genes is comprised of 35 known genes and 9 “predicted” genes (Tables 2 and 3). The predicted genes (*Gm1269, Gm3917, Gm1268, Gm6856, Gm7000, Gm8613, Gm4260, Gm2019* and *Gm5770*) have not been further studied. Of those tumors that had at least 1 MMTV RIS, approximately 58% also had one or more candidate MMTV CIS. This rendered tumors not having a CIS insertion difficult to interpret as containing any transforming mutation. The most frequent genes affected by MMTV CIS were members of the *Wnt, Fgf*, and *Rspo* gene families (Table2) and have been designated as the “Core” CIS genes.

A group of 8 MMTV CIS genes was identified where the viral integration events all occurred within the gene, including: *Notch4, Nckap5, Pde3a, Usp31, Nxn, Phactr1, Cadm2* and *Kcnj6* (Table 3). Except for *Nckap5, Pde3a* and *Kcnj6* all of the genes are expressed in the normal mammary gland of pregnant mice [17, 18] and data not shown. *Kcnj6* expression is not detected in tumors in which it is rearranged by MMTV. We have not exhaustively surveyed the mammary gland RNA for *Kcnj6* mRNA, therefore, we cannot exclude the possibility that there is a small window of time during mammary development when Kcnj6 is expressed.

The transcriptional orientation of the MMTV genome relative to the transcriptional orientation of the gene in which it integrated, was determined for four genes (*Usp31, Nckap5, Cadm2* and *Notch4*) for which there was available tumor RNA. We have found that in each case the proviral genome is in the same transcriptional orientation as the target gene and is associated with the transcription of a chimeric RNA initiated from the U5 region of the 3' MMTV LTR through host sequences 3'of the viral integration site. The RT-PCR products using nested MMTV LTR U5 forward and reverse primers from an exon 3' of the integration site are shown in Figure 1. In Table 4, the introns in which the intragenic integration events occurred and the location of the next in-frame translation start signal 3' of the MMTV integration site are given. The biological activity of these truncated mRNA transcripts will be the subject of a future study.

**Table 4:** Transcriptional Orientation of MMTV in MMTV CIS Gene^1^

Gene	CIS	MMTV Orient.	Translation Start Signal EXON	In frame ATG bp of mRNA
Nckap5	Intron 6	same	7	719
Pde3a	Intron 1	NT	2	1279
Usp31	Intron 15	same	16	2566
Nxn	Intron 2	NT	5	829
Phactr1	Intron 3	NT	4	629
Cadm2	Intron 1	same	4	1019
Notch4	Exon 22	same	24	4151

1 The transcriptional orientation of MMTV in intrgenic MMTV CIS genes. The intron or exon in which MMTV integrated is in the CIS column. The transcriptional orientation of the virus relative to the transcription orientation of the gene is indicated. The exon 3' of the MMTV integration site containing the next in-frame translation initiation site and its location in the mRNA is indicated. NT = not tested.

*Notch4* viral integration events all have occurred within the coding portion of the gene and result in the transcription from the 3' MMTV LTR of the region encoding the intracellular domain of the receptor protein[19]. The *Notch4* MMTV integration events represent gain of function mutations. In the present study no MMTV insertions were detected in *Notch4* in the panel of CzechII tumors. However, 1 Balb/cfMMTV^CZ^ and 2 Balb/cfMMTV^SP^ mammary tumors had MMTV insertions within *Notch4/Int3*. Previously *Notch4/Int3* was reported to be a CIS in 2 Balb/cfMMTV^C3H^, 2 BR6 mammary tumors and in 43% of feral *Mus musculus jyg* mammary tumors [12, 20, 21].

### MMTV CIS genes in premalignant lesions, primary tumors and metastases

**Figure 2:**
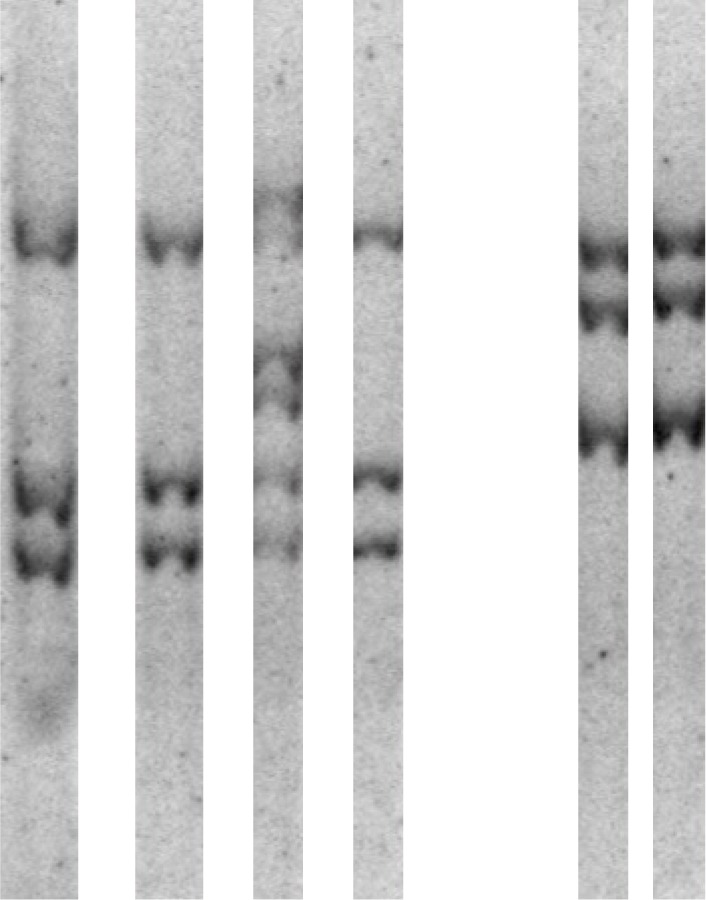
Southern blot of EcoRI digested DNA from: Lane 1, Czz26 mammary tumor (MT)-12; Lane 2, Czz26-MT12-metastasis (Met)-A; Lane 3, Czz26-MT12-MetB; Lane 4, Czz26-MT12-MetC; Lane 5, Czz28-MT6/7; and Czz28-MT6/7-Met The blot was probed with ^32^P labeled MMTV envelope (ENV) specific probe.

MMTV from feral CzechII mice induces transplantable preneoplastic HANs that can be maintained indefinitely as mammary premalignant HOG lines by transplantation into epithelium-divested CzechII mammary fat pads (reviewed in [2]). Within these populations pregnancy independent mammary tumors develop stochastically. Premalignant mammary HOGs, HOG derived tumors and lung metastases as well as spontaneous MMTV induced mammary tumors were analyzed to determine whether linkage exists between particular MMTV CIS genes and stage of tumor development. *Rspo2*, but not *Rspo3*, was a MMTV CIS in 10 out of 15 independent CzechII HOGs (Table 2). In this series of HOG DNAs, one had a viral insertion at *Pdgfra*. These results extend an earlier smaller study in which the MMTV CIS genes; *Rspo2, Wnt1* and *Fgf3/4* were each found to be activated in CzechII HOGs [13]. Out of 90 HOG derived mammary tumors that were analyzed, only 6 had a MMTV CIS in addition to the one observed in the precursor HOG. These CIS genes include: *Pdgfra*, *Fgf3*, *Rspo2, Wnt1* and *Col4a5* (Table 2). Similarly, only one of 15 independent lung metastases from 11 mice had an MMTV CIS in addition to those in the precursor HOG or mammary tumor. Figure 2 shows a Southern blot of EcoR1 restricted cellular DNAs from mammary tumors Czz26MT12 and Czz28MT6/7 and matching lung metastases. The lung metastases occur as individual lung nodules in the lung of a tumor-bearing host. Each band corresponds to an acquired viral genome. With the exception of one Czz26MT12 lung metastasis B (Figure 2, Lane 3) there was no additional viral integration events in the lung metastasis compared to the matching tumor. Our analysis suggests that each lung metastatic nodule is an independent clone (Figure 2, lane 3). The three additional MMTV integration events in one of the nodules suggests that these events had little to do with the acquisition of a metastatic phenotype since all of the nodules were from the same mammary tumor and formed in the lung of the same host.

### Effect of MMTV integration on the expression of clusters of closely linked genes

In addition to 19 unlinked MMTV CIS genes, there are 4 MMTV CIS loci comprised of 11 genes in total (Table 2). One locus is composed of three linked “predicted” genes and has not been studied further. Most of the MMTV-activated genes are not expressed or are expressed at very low levels in normal mammary epithelium. The molecular basis for virally induced gene activation is not well understood, but seems to be linked to the effect of transcription enhancers in the U3 portion of the MMTV LTR on the target gene's transcriptional promoter. The distance between the activating MMTV RIS and the target CIS gene ranged from 100 bp to greater than 200Kb (See Supplementary Figure 1), for example: *Rspo3*, 246Kb (Tumor#5447); *Phf19*, 663 Kb (Tumor#6749) and *Tacstd2*, 716Kb (Tumor#7856). Therefore gene expression activation appears to occur over significantly greater linear distance from MMTV integrations than previously reported [10, 12]

MMTV integration near the *Wnt1/Wnt10b* and *Fgf3/Fgf4* loci can lead to the expression in the same tumor of one or the other or both genes in these clusters [12, 20, 22]. As shown in Table 5 and Supplemental Figure 2, *Fgf15* on chromosome 7 is 31Kb 3' of *Fgf4* and is frequently activated by MMTV integration events occurring 5' of *Fgf3*, between *Fgf3* and *Fgf4*, and between *Fgf4* and *Fgf15*. On chromosome 15, MMTV integration events occurring in *Arf3*, between *Wnt10b* and *Wnt1* or in *Wnt1* are associated with the activation of expression of *Ddn* that is 14Kb 3' of the *Wnt1 locus*. Likewise, MMTV integration 5'of *Fgf8* on chromosome 19 is associated with activation of its expression and frequently the expression of *Npm3* which is located 13Kb 3' of *Fgf8*. A similar phenomenon has been observed in rat T-cell lymphomas by Moloney murine leukemia virus integration at *Mlvi-1* or *Mlvi-4* on the activation of expression of *Myc* [23].

**Table 5:** Clusters of MMTV CIS genes^1^

Chromosome 7								
Distance (bp)			18,398 bp		31,289 bp			
Tumor ID		Fgf3		Fgf4			Fgf15	
3369	I	N		Y			Y	
3430	I	N		N			Y	
3955	I	Y		N			Y	
3997		N	I	N			Y	
4114		N	I	Y			Y	
4342	I	N		N			Y	
5262		Y		N	I		Y	
5294		Y		Y	I		Y	
5447	I	N		Y			Y	
6305		N	I	N			Y	
6463		N	I	N			Y	
**Chromosome 15**								
**Distance(bp)**		**34,139 bp**		**18,095 bp**			**13,920 bp**	
**Tumor Id**	**Arf3**		**Wnt10b**			**Wnt1**		**Ddn**
3997	N		N			N		Y
4114	N		N	I		N		Y
4387	N I		N			Y		Y
4533	N		N			Y 2 I		N
6638	N I		Y			Y		Y
7454	N		N	I		Y		Y
4342	N		N			Y I		Y
4354	N		Y			Y I		Y
4443	N		Y	I		Y		Y
4475	N		N	I		N		Y
6385	Y		Y	I		Y		Y
**Chromosome 19**								
**Distance(bp)**			**12,745 bp**					
**Tumor ID**		**Fgf8**		**Npm3**				
6638	I	Y		Y				
6305	I	Y		Y				
8000	I	Y		N				
4485	I	Y		Y				
3430	I	Y		N				
3369	I	Y		Y				
4143	I	Y		Y				

1 Three examples of the effect of MMTV integration on the expression of clusters of genes. The distance between the genes is indicated in base pairs. Genes that are expressed are indicated with “Y” and those not expressed with “N”. “I” indicates the region of the cluster in which MMTV integrated ie. 5'of *Fgf3*, in between *Fgf3* and *Fgf4* or *Fgf4* and *Fgf15*. In some tumors the virus integrated within *Wnt1*.

### Expression of MMTV CIS gene orthologues in human breast carcinomas

The Oncomine Database [24, 25] was interrogated for expression of the 26 human orthologues of the MMTV CIS genes (Tables 2 and 3). In the Radvanyi [26] study genes were grouped on the basis of their ranking in the top 5-10% of genes in the particular type of breast carcinomas where expression was detected (Table 6). NCKAP5 expression was found in invasive ductal carcinomas (IDC), mixed IDC with ductal carcinoma *in situ* (DCIS) and invasive lobular carcinoma (ILC) expression patterns. Likewise, PHF19 is frequently up regulated in several types of cancer [27] including human breast IDC, ILC and DCIS and is associated with tumor progression (Table 6). FGF19 was expressed in mixed IDC/DCIS and ILC (Table 6). FOXL1 was highly expressed in DCIS and ranked in the top 5% of genes analyzed in the study (Table 6).

**Table 6:** Expression of MMTV CIS gene orthologues in human breast tumors^1^

Invasive Ductal				
Gene Symbol	Median Rank	p-value	Fold Change	Rank
NCKAP5	1030	0.02	2.863	10
PHF19	763	0.013	2.462	10
**Invasive Mixed**				
NCKAP5	326	0.005	4.132	5
PHF19	1327	0.024	2.2	25
**Invasive Lobular**				
NCKAP5	1447	0.038	2.54	10
PHF19	292	0.007	2.863	10
SFMBT2	4009	0.121	2.5	25
FGR	3266	0.094	1.621	25
FGF19	1056	0.027	2.856	10
DDN	3754	0.112	3.382	25
**Ductal Carcinoma In Situ**				
NCKAP5	2194	0.084	2.474	25
PHF19	763	0.01	2.627	10
FOXL1	2154	0.055	4.33	5

1 The human orthologues of the MMTV CIS genes were evaluated in the Oncomine database [24, 25]. In the Radvanyl [26] study a collection of invasive ductal (IDC), invasive mixed (IDC and with ductal carcinoma *in situ* (DCIS)), invasive lobular carcinoma (ILC) and DCIS were evaluated for the expression of the CIS genes. The Median Rank for a particular gene across all of the analysis is indicated as well as the p-value of the gene for the median rank analysis is also given. The Rank number (1, 5, 10 or 25) of a gene indicates that it is in the top 1%, 5%, 10% or 25% of genes in that study. Fold change is relative to normal tissue.

### MMTV CIS gene orthologues that are high-risk genes in human cancer

As a further test for consistency between our MMTV CIS genes and genes identified in human breast cancer the human orthologues corresponding to the list of 27 MMTV CIS genes found in MMTV induced tumors (Table2) were compared with lists of genes in The Cancer Gene Atlas (TCGA) [28, 29]. Using the TCGA Gene Ranker [29] we have found that 11 out of the 26 genes had a score equal to or greater than 1.0 and a ranking of 11 to 3362 out of 7658 genes, making them “high-risk genes” in cancer (Table 7). For instance *Sdc2* is rated as a “high risk” gene in the TCGA. Of the high-risk genes, 6 were also found in the Broad [29], Cosmic [30], Sanger [31] and Volgestein/Kinzler [32] databases of human tumor-associated mutations.

### Expression of newly found MMTV CIS genes in non-tumorigenic HC11 mammary epithelial cells

**Figure 3:**
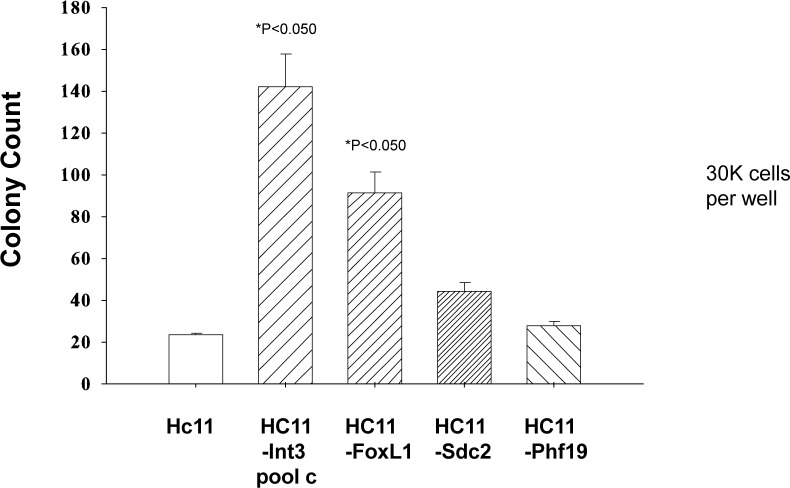
Anchorage independent-growth of HC11 cells stably expressing *Foxl1*, *Sdc2* or *Phf19* in soft agar HC11-*Notch4/Int3* cells were a positive control (*Int3* is the “activated form of *Notch4*) and HC11 cells were a negative control. Soft agar was seeded with 30,000 cells and colonies were counted after three weeks of incubation. The number of colonies is indicated on the Y-axis and the cell line is indicated on the X-axis. The number of colonies for HC11-Int3 and HC11-Foxl1 are significantly (*P*<0.05) greater than for HC11.

**Figure 4:**
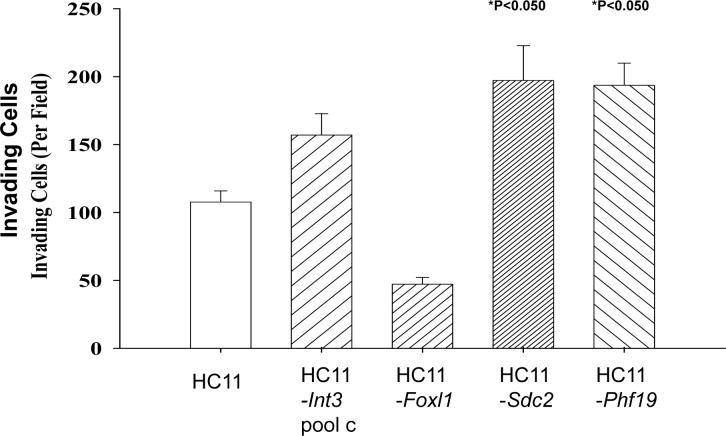
Invasion assay of HC11-*Foxl1*, -*Sdc2* and -*Phf-19* cells Cells (10,000) were seeded in the top Boyden chamber and incubated for 48hr. HC11 and HC11-*Int3* cells were tested for comparison. The number of invading cells is indicated on the Y-axis and the cell line is indicated on the X-axis. The number of invading cells for HC11-Sdc2 and HC11-Phf19 are significantly (*P*<0.05) greater than for HC11.

The consequences of Core MMTV CIS gene expression in transgenic models on mammary gland development and tumorigenesis as well as in tissue culture with mammary epithelial cells is well documented (reviewed in [2]). To further validate an association between MMTV CIS genes and mouse mammary tumor progression, three low frequency MMTV CIS genes (*Phf19, Foxl1* and *Sdc2*) were selected for further study to determine whether their expression confers on mammary epithelial cells the capability for anchorage independent growth and/or invasion on mammary epithelial cells. The rationale for picking *Phf19* and *Foxl1* was that their expression was frequently deregulated in primary human breast carcinomas (Table 6). *Phf19* encodes a member of the polycomb group of proteins that function by maintaining repressive transcriptional states of many developmental regulatory genes throughout embryogenesis and into adulthood [27]. *Foxl1* is a transcription factor [33] that is a regulator of *Wnt5a* transcription [34]. *Sdc2* is at high risk of being mutated in human cancers (Table 7). *Sdc2* plays a critical role as an adhesion receptor during cancer cell migration [35, 36]. HC11 mouse mammary epithelial cell lines stably expressing *Phf19, Sdc2* or *Foxl1* RNA and protein (Supplementary Figures 3-5) were tested for anchorage independent growth in soft agar. As quantified in Figure 3, HC11-*Foxl1* cells (30,000) were capable of forming colonies in soft agar where as HC11-*Sdc2* and HC11-*Phf19* cells were not. In another assay measuring the ability of the cells to penetrate Matrigel and migrate through a pore in the underlying membrane of a Boyden Chamber, HC11-*Phf19* and -*Sdc2* cells (10,000 each) were invasive (Figure 4) whereas HC11-*Foxl1* cells were not. HC11-*Phf19*, -*Foxl* and -*Sdc2* cells were also injected subcutaneously into the mammary fat pad of athymic nude mice to test for their tumor inducing capability. After five weeks tumors were palpable in 4 out of 6 mice injected with 2 × 10^6^ HC11-*Foxl1* cells. No tumors were detected after 5 weeks in 6 mice each injected with 2 × 10^6^ HC11, HC11-*Sdc2* or HC11-*Phf19* cells, respectively.

**Table 7:** CIS gene orthologues in human tumors^1^

Mouse or Virus Strain	Gene ID	Human Gene Symbol	Gene Ranker Score	Rank out of 7658	Broad	Cosmic	Vogelstein	Sanger
MMTVSP	2268	FGR	1.25	2587	Y			
Core	5156	PDGFRA	11	11	Y	Y	Y	Y
Core	2248	FGF3	1.25	2585				
Core	2249	FGF4	1.25	2586				
MMTVSP	2E+05	PHACTR1	1.5	2319	Y			
MMTVCZ & SP	6383	SDC2	2	1741				
Core	3E+05	RSPO2	1.5	2383		Y		
Core	7480	WNT10B	2.5	980		Y		
Core	7471	WNT1	1.25	2829	Y			
MMTVCZ & SP	4855	NOTCH4	2.75	740	Y			
Core	2253	FGF8	1.5	2141				

1 The human orthologues of MMTV CIS genes that are at risk of mutation in human cancers as determined by The Cancer Gene Atlas (TCGA) [28, 29]. TCGA is comprised of 39 gene lists containing a total of 7658 genes which have been ranked in part by the number of gene lists on which they appear. Each list that is contributed to Gene Ranker gets a score: [38] and a ranking out of the 7658 genes in the TCGA catalogue. A ranking of less than 3500 is associated with a gene that is at “high risk” for mutation in human cancer. A “Y” indicates that mutations have been found in that particular gene in the Cosmic [30], Sanger [31], Broad 2000 [29] and/or Volgestein/Kinzler [32] databases of mutations identified by nucleotide sequence analysis. The particular mouse or virus strain with which the gene was identified is indicated.

## DISCUSSION

### Orthologues of MMTV CIS genes are deregulated or mutated in human breast cancer

One important goal of the present study was to identify genes which when activated/inactivated by MMTV contribute to mammary tumor development in mice and thus represent candidates for contributors to malignant progression in human breast tumors. Three MMTV CIS genes (PHF19, FOXL1 and SDC2) were shown to confer properties associated with malignant transformation in HC11 mammary epithelial cells. FOXL1 and PHF19 were also found to be highly expressed in breast DCIS and IDC, ILC and DCIS, respectively. SDC2 was listed as a high-risk gene for cancer in the TCGA database. In addition, the expression of NCKAP5 and FGF19 is deregulated in specific histopathological types of human breast tumors (Table 6). The function of NCKAP5 is unknown. FGF19 is the human orthologue to mouse gene, *Fgf15*. Eleven of the CIS genes (Table 7) were ranked as high-risk genes in cancer and 3 of these genes (PDGFRA, RSPO2, and WNT10B) were mutated in human tumors. In addition, Dr. Bert Vogelstein (unpublished data) has found that 8 of the 27 CIS genes have been shown to have non-synonymous mutations in at least one human tumor, which approaches statistical significance (p =0.05, Chi^2^) (Personal communication). It is important to point out that inbred and closed colony mice are representative of a rather small and limited genomic diversity whereas the human population represents a much broader genomic diversity. Thus commonalities in gene pathways involved with mammary carcinogenesis in the human population may be considered to have a greater scientific significance than comparisons between individual breast tumor transcriptomes.

### MMTV CIS are initiating mutations

MMTV integration events do not appear to be a driving force in the transition from a pre-malignant lesion to malignant tumor to metastasis. Only some of the HOG-derived mammary tumors and one of the metastases had additional MMTV CIS compared to the preceding stage of tumor progression. This is reminiscent of an earlier study comparing C3H mammary tumors with C3H HOGs and HOG derived tumors [37]. In that study *Wnt1* and *Fgf3* were activated by MMTV in 52% and 14% in C3H mammary tumors, respectively where as in the HOG derived tumors they were activated in 6% and 0%, respectively. The observation that MMTV can super-infect MMTV ENV expressing cells suggests that the role of MMTV CIS is in the initiation of pre-malignant mammary lesions or mammary tumors and that alternative somatic mutations are more likely responsible for tumor progression [38]. Similarly the pregnancy-dependent “Plaques” may be induced by MMTV^SP^ activation of *Fgr* or *Phactr1* but other non-viral related events lead to pregnancy independence. Alternatively MMTV^SP^, like MMTV^CZ^, may activate a common set of low frequency CIS that initiate pregnancy independent mammary tumors. This model does not rule out collaboration between different MMTV CIS genes. For instance pre-malignant mammary HOGs frequently contain MMTV CIS at *Rspo2* and *Wnt1* or *Fgf3* [13]. In favor of *Rspo2* collaborating with *Wnt1*, *Rspo2* has been shown to amplify β-catenin dependent Wnt1 signaling [39]. The observation in *Wnt1/Fgf3* bitransgenic mice that mammary tumor development is a stochastic event is consistent with the necessity for collaborating events during mammary tumor progression [40].

### Demonstration of the biological consequences of Sdc2, Phf19 and Foxl1 expression on mammary epithelial cells

Sixteen CIS genes were identified in CzechII, Balb/cf MMTV^CZ^ and Balb/cf MMTV^SP^ mammary tumors that occur at a low frequency and are mouse or virus strain specific. For two of these genes (*Sdc2* and *Phf19*) the up regulation of their expression in HC11 cells confers on them invasive properties but not the capability for anchorage independent growth in soft agar or ectopic growth in athymic nude mice. This raised the possibility that the selection for *Sdc2* and *Phf19* expression during mammary tumorigenesis is a collaborative event with other MMTV activated genes in the same tumor. In each of the tumors in which MMTV activated *Sdc2* or *Phf19*; MMTV also activated *Wnt1*, *Fezf1*, *Sfmbt2*, *Ddn*, *Rspo2*, *Rspo3* or *Fgf3*. Similarly Kim et al. [10] have shown that *Tcf7l2*, *Antxr1/Tem8* and *Arhgap18* MMTV CIS, which appear at a low frequency, confer on cells altered growth kinetics and morphological transformation in three dimensional culture and occur in mammary tumors having multiple MMTV CIS. Expression of *Foxl1* confers on HC11 cells the capability for anchorage independent growth in soft agar and also tumor formation in athymic nude mice. It is worth noting that both alleles of *Trp53* are mutated in HC11 cells.

The present study supports the importance of studying mammary tumorigenesis in the mouse as a means to a better understanding of breast cancer and its causes in the human. These data indicate a similarity between events seminal to MMTV-induced mammary tumorigenesis and those involved in sporadic human breast carcinogenesis. Though the number of mutations in these genes or their deregulated expression is not enough to classify them as drivers of malignancy, our work provides functional evidence that could be used to prioritize them for further study in the future. Development of mouse models in which the MMTV CIS gene (*Wnt1, Wnt10b, Fgf3, Fgf4, Notch4* and *Eif3e*) is expressed as a transgene in the mammary gland has proven to be a valid approach. These models have confirmed the involvement of these genes in mouse mammary tumorigenesis and represent an experimental approach for the future studies of the MMTV CIS identified here.

## MATERIALS AND METHODS

### Mouse strains

The feral *Mus musculus musculus* CzechII (CZ) and *M. spretus* (SP) mouse strains have been described previously [9]. The development of CzechII preneoplastic mammary outgrowth lines (HOGs) and their properties is described in the Supplementary Methods. In this study CzechII, Balb/cfMMTV^CZ^ and Balb/cfMMTV^SP^ pregnancy independent mammary tumors as well as CzechII HOGs, HOG derived tumors and specific lung metastases were analyzed. The numbers of tumors analyzed and the number of retroviral integration sites (RIS) identified in each is presented in Table1.

### Southern blot analysis

Total DNA from HOG derived mammary tumors and selected matching lung metastases in the same animal was prepared and digested with EcoRI as previously described [41]. Restriction fragments were electrophoretically separated on an agarose gel and transferred to a membrane as previously described [41]. The subsequent blot was hybridized with ^32^P labeled MMTV envelope (ENV) specific probe.

### MMTV host-viral junction fragments

MMTV integration junction sites were cloned using inverse polymerase chain reaction (PCR) as described previously [13, 42]. Briefly, CzechII, Balb/cMMTV^CZ^ and Balb/cfMMTV^SP^ mammary tumor genomic DNA was digested with the restriction enzyme cocktail of Bgl II, Bcl I and BamHII. This product was diluted to reduce random concatenation of the digested fragments and self-ligated into closed circles. The junctions of the host-viral integration sites were PCR amplified using a nested system of two primer pairs (see Supplementary Methods for the conditions and primers).

### Identification of MMTV RIS and MMTV common integration sites (CIS)

A MMTV RIS corresponds to the site in the host genome in which the viral genome has integrated. A cluster of MMTV RISs, which affect the expression of the same gene or adjacent genes, corresponds to a MMTV common integration site (CIS). A total of 5288 clones of MMTV-host DNA fragments from 345 independent CzechII, Balb/cMMTV^CZ^ and Balb/cfMMTV^SP^ mammary tumors were sequenced. These sequences were then mapped to mouse genome version 9 using the BLAT program (UCSC genome server, [43]). Redundant sequences were removed for each tumor. For identifying MMTV CIS or clusters of MMTV RIS near a gene, we used the statistical analysis procedure describe by Mikkers et al. [15]. Calculation of CIS clusters was done at the website: [44]. It has a unique tool developed to cluster viral insertion data based on the number of insertions and then obtain genes that are closest to these CIS clusters. The tool was developed using scripts that calculate distances for determining insertion clusters based on the statistical analysis described earlier [15]. The number of total insertions spread across the genome influences the likelihood that any 2 insertions will be observed within a given distance. For 591 insertions and a probability of 0.01, we determined this distance to be 46 kb. Similarly for three or more insertions to be considered clustered they have to occur within a distance of < 650kb (Table1). Since many genes can fall within this window, we chose the closest genes to each of the clusters, any genes with intra-gene insertions or within a very close distance (<2000 bases) from any sequence within the cluster as a CIS gene. These distances are based upon a linear evaluation of the DNA, it is not known exactly how close to each other the insertions and the genes affected are in the compacted chromatin of the affected cell. The list of CIS genes generated in this manner was compared using our visualization tool [21] to look at the microarray expression results ordered by chromosome location (see below). For each of the CIS genes in the primary list the adjacent genes were examined to determine any changes in their expression. Genes that were positive for expression by microarray analysis were selected for confirmation by RT-PCR analysis of RNA from tumors that were in the microarray analysis as well as those that were not in that analysis that had a viral integration event near or within the particular gene. The final CIS gene list was then compiled from the results of all these steps.

### RNA extraction, microarray and quantitative RT-PCR analysis

HC11 mouse mammary epithelial cells were grown to 80% confluence, trypsinized, washed with 1xPBS, and pelleted for lysis. Total RNA was made using the RNeasy Mini Kit (Qiagen) per manufacturer's instructions. Total mammary tumor RNA for microarray analysis or quantitative reverse transcriptase (RT) PCR was prepared using TRIzol reagent (Invitrogen Life Technologies, Carlsbad, CA, USA) followed by treatment with RQ1 DNAse-I (Promega, Madison, Wisconsin), according to the manufacturer's recommendations. The quality and quantity of RNA was measured by Agilent bioanalyzer-2100 (Agilent Technologies, CA, USA) according to the manufacturer's instructions. Mammary tumor or mammary tissue RNA from a day 15 pregnant mouse (as a control) was hybridized to Affymetrix GeneChip HTA Mouse Genome 430A and 430B Arrays. Quantitative RT PCR was performed using the Brilliant II SYBR Green QRT-PCR Master Mix 1-Step Kit (Agilent Technologies) per manufacturer's instructions. Samples were run in the Stratagene Mx3000 pro cycler, set up in duplicate and normalized against *Gapdh* using the Mxpro software.

### Visualization of microarray results

The gene expression results from our microarray experiments were loaded into an Oracle 11g database. This data was then combined with the mouse gene chromosomal location information obtained from NCBI and the ordered result set, ordered by genomic location, is displayed as a series of colored bars in a tabular format [16].

### Preparation of cDNA products from tumor RNA

The cDNA was prepared from 1μg of total cell or tumor RNA using the Superscript One-Step RT-PCR kit from Invitrogen (Carlsbad, CA) with Platinum Taq Polymerase per reaction according to the manufacturer's instructions. The primers used are given in Supplementary Table 1. The products were electrophoretically separated 0.8% agarose gels.

### Verification of MMTV CIS within a gene

The site of MMTV integration in the intra-gene CIS was verified by analysis of RT-PCR products spanning the host-viral junction mRNA sequences from MMTV induced mammary tumor RNA using the Invitrogen “One Step RT PCR with Platinum Taq” kit and the manufacturer's reaction conditions. The PCR fragments were obtained using MMTV LTR U5-specific primer and a reverse primer from an exon 3' of the viral integration site (Supplementary Table 2). The RT-PCR products were electrophoretically separated on a 2% agarose gel. The DNA bands were cut out from the gel and purified using the Qiagen gel purification kit. The fragments were re-amplified using nested primers (Supplementary Table 2) and re-purified by gel electrophoresis. No RT-PCR products were obtained using forward primers from exons 5' of the integration site and reverse primers from the MMTV LTR in either viral transcriptional orientation (data not shown).

### HC11 mouse mammary epithelial cells transfected with EF6-*Phf19*, *Sdc2* or *Foxl1*

Three Open Reading Frame cDNA Clones were purchased (Origene, TrueClone ORF Collection), each containing *Foxl1*, *Sdc2* and *Phf19*, respectively. Each vector was digested with EcoR1 and Not1 and the open reading frame of the respective gene was cloned into the pEF6/V5-His Vector (Invitrogen) at the EcoR1/Not1 sites. HC11 mouse mammary epithelial cells cultured in RPMI 1640 media containing 10% fetal bovine serum, 2 mM glutamine, 5 μg/ml insulin and10 ng/ml of mouse recombinant EGF were split the day before transfection at roughly 1×10 ^5^ cells/ml into 6 well microtiter plates to establish 70% - 80% confluency on the day of transfection. For each well, 1 ml of serum free media was placed in a separate reaction tube with 1 μg DNA followed by 3 μl GenJet Reagent II (SignaGen Laboratories) was added to the reaction tube gently mixed and incubated at room temperature for 20 minutes mixture was then added drop-wise onto the HC11 cells containing fresh HC11 media. The cells were incubated 48 hours at 37°C in 5% CO2 followed by selection media containing 10 μg/ml Blasticidin. Pools of cells were selected and RNA was prepared to perform quantitative RT PCR to assess gene RNA expression levels. Western blot analysis with protein specific antibodies was used to assess protein expression.

### Protein Extraction and Western blot analysis

HC11 cells were harvested and protein was made using the NE-PER Nuclear and Cytoplasmic Extraction Reagents (Thermo Scientific) per the manufacturer's instructions. Nuclear and Cytoplasmic proteins were quantitated using the Nanodrop (Thermo Scientific) to measure absorbance at 280 nm and 50 ug were mixed with 2x Loading Dye, denatured at 99° for 5 minutes and run on a Tris-Glycine gel (Invitrogen). Protein samples were run on a 4-20% Tris-Glycine gel (Invitrogen) at 115 volts for 2 hours and transferred to a PVDF membrane (Invitrogen) using the manufacturer's instructions. Membranes were blocked overnight at 4°C in 5% milk TBS-T buffer (Tris-buffered saline, pH 7.4 with 0.05% Tween 20). Primary antibody Anti-V5 (Invitrogen) was diluted at 1:4000 in 5% milk TBS-T buffer and incubated at room temperature with rocking for 2 hours. The membrane was then washed 4 times, 15 minutes each with TBS-T buffer. The secondary antibody, anti-mouse conjugated to horseradish peroxidase (GE Healthcare /Amersham) was diluted 1:5000 in 5% milk TBS-T buffer and incubated at room temperature with rocking for 1 hour. The membrane was washed 4 times with TBS-T, 15 minutes each and a final wash with TBS buffer. Membrane staining was developed using chemiluminescent reagents provided in the ECL kit (GE Healthcare / Amersham) according to manufacturer's instructions. Bands were visualized on film using varying exposure times.

### Soft agar assay for anchorage independent growth of HC11 cells

Soft agar colony growth was conducted as previously described (Raafat et al 2007. Final cell concentrations in the agar mixture were 15,000 or 30,000 cells/ml per well, plated in triplicate for each stable cell line and plates were incubated at 37°C with 5% CO2 for 3 weeks. To count colonies, wells were stained overnight at 37°C with 500 μl Nitrobluetetrazolium (NBT) at 1 mg/ml in 1X PBS. Stained colonies were counted using the AccuCount 1000 automated colony counter system (BioLogics, Inc, Manassas, VA, USA).

### Invasion assay of HC11 cells

Invasion was measured using 24-well cell culture inserts with membranes having 8-μm pores and a Matrigel-coating (BD Biosciences; San Jose, CA). Cells were suspended in serum-free medium with 0.1% BSA at a concentration of 20,000 cells/ml. 500 μl of diluted cells were plated in the top part of the insert. The inserts were placed in wells containing complete HC11 medium and incubated at 37°C for 48 hours. Residual cells were wiped off of the top of the membranes with cotton swabs, and invaded cells on the underside of the membranes were fixed and stained with Diff-Quik Stain Set (Siemens). Cells were counted from 3 membranes per experimental condition. Experiments were performed in a minimum of two independent studies.

### Tumor formation by HC11 cells expressing MMTV CIS genes

Nulliparous 3 week-old athymic nu/nu female mice were purchased from NCI/Frederick and used at 10 weeks of age as hosts for the transplantation study. The inguinal mammary glands of these nude mice served as the injection site of the cell suspensions. In brief, the mice were anesthetized, and the cell suspensions (2 x10^6^) in 10 μl of PBS were mixed with 10 μl of 2X Matrigel (BD Biosciences) and injected with a Hamilton syringe equipped with a 30-gauge needle. Five cell lines were tested, HC11, HC11-*Notch4/Int3*, HC11-*Foxl1*, HC11-*Sdc2* and HC11-*Phf19*. Six females were used for each cell line, to maximize the use of the mice, both inguinal mammary glands number 4 and number 9 were injected with 2 x10^6^ cells each/mouse. The implanted females were maintained nulliparous. Females were palpated twice every week. All mice were housed in Association and Accreditation of Laboratory Animal Care-accredited facilities in accordance with the NIH Guide for the Care and Use of Laboratory Animals. The National Cancer Institute Animal Care and Use Committee approved all experimental procedure.

### Statistics

Quantitative values are represented as the mean of at least three experiments. The stastistical significance of the difference between groups was determined by the Wilcox rank sum test. Comparisons resulting in *P-*values less than 0.05 were considered statistically significant and identified in the figures with an asterisk (*).

## Supplementary Tables, Methods, and Figures






