# Supplementary material for: Genes affected by mouse mammary tumor virus (MMTV) proviral insertions in mouse mammary tumors are deregulated or mutated in primary human mammary tumors
Source: Oncotarget. 2012 Oct 18;3(11):1320–34. doi: 10.18632/oncotarget.682 (PMC3717796; doi:10.18632/oncotarget.682)
Supplement: Supplementary file 2 [file oncotarget-03-1320-s002.pdf]

Genes affected by mouse mammary tumor virus (MMTV) proviral insertions in mouse mammary tumors are deregulated or mutated in primary human mammary tumors - Callahan et al

Supplementary Methods

Mouse Strains

Hyperplastic alveolar nodules (HAN), which were subsequently transplanted into epithelium-free mammary fat pads, were readily found in the exposed mammary glands of retired CzechII breeders whether they were tumor-bearing or not. Characterization of the hyperplastic nature of these lesions was ascertained at the end of the 1st transplant cycle. Only those lesions that produced clear hyperplastic outgrowths (HOGs) were maintained by serial passage. Each HOG line thus established displayed a mammary tumor incidence unique to that particular line. All of the lines showed an incidence of stochastic mammary tumor development that was greater than that observed among breeding CzechII females. Some of the mammary tumors arising in these HOG lines produced metastatic lung and splenic lesions. These were collected and transplanted into gland-free mammary fat pads to demonstrate their tumorigenic capacity. In some instances multiple lung metastases were found in an individual mouse. These all produced tumors upon transplantation and were found to be independent clonal populations. The *M.spretus* (SP) mouse strain was developed with 4 founders trapped in Puerto Real, Spain [9]. This mouse strain has a 20% incidence of pregnancy dependent mammary tumors that in subsequent parities become pregnancy independent. MMTV from each of these mouse strains was transmitted to Balb/c inbred mice by foster nursing on CzechII or *M.Spretus* lactating females. In this study CzechII, Balb/cfMMTV^{CZ} and Balb/cfMMTV^{SP} pregnancy independent mammary tumors were analyzed.

MMTV host-viral junction fragments

Host-viral junction fragments from CzechII, Balb/cfMMTV^{CZ} and Balb/cfMMTV^{SP} mammary tumor DNA was amplified using one primer set in the LTR (LTR5-100-r1) the other in the GAG region (GAG-2720-f1). The LTR primers face 5' and the GAG primers face 3'. A second round of PCR was performed with primers designed in a manner similar to the 1st, using GAG-2800-f2 and LTR5-40-r2 and the resulting junction site sequences were cloned directly into the TOPO-TA vector (Invitrogen, CA). The nucleotide sequence of 48 clones from each tumor source was determined by the Sanger method from the M13 site in the vector. The MMTV LTR primers used in this study include GAG-2720-f1 (CCTCCTGGAGTTAAA AAGACTGT ATTAGC), LTR5-100-r1 (CGGGTGCACGCAGACGGGTCG TCCTTGG), GAG-2800-f2 (CATTTCAAGGCTCGAGGAAGCTGTTTACAG), LTR5-40-r2 (CCTAAGTG TAGGACATCTCGGGAGTTC).

Oncomine gene rank

The data-mining platform Oncomine, allows for assessing differential expression of selected genes across many data sets. In each of these data sets the genes studied are ranked top to bottom based on their significance as seen by the differential expression analysis [24, 25].

The Cancer Genome Atlas (TCGA) database [28]

TCGA is comprised of 39 gene lists containing a total of 7658 genes which have been ranked in part by the number of gene lists on which they appear. Each list that is contributed to Gene Ranker gets a score. You can see the scores on line at: [29]. For the purpose of selecting genes for the next phase of TCGA sequencing (which this resource was used for), a score of 1.0 was assigned to lists of genes (Cosmic [30], Sanger [31], Broad 2000 [28] and Volgestein/Kinzler [32] databases) that were shown to be mutated in particular tumors, other gene lists were assigned a somewhat lower weight (0.5 or 0.25, for example for genes derived from expression studies or signaling pathways - these gene lists are typically larger). Only genes with a score of 1.0 or greater are selected for sequencing.
