# Supplementary material for: Genes affected by mouse mammary tumor virus (MMTV) proviral insertions in mouse mammary tumors are deregulated or mutated in primary human mammary tumors
Source: Oncotarget. 2012 Oct 18;3(11):1320–34. doi: 10.18632/oncotarget.682 (PMC3717796; doi:10.18632/oncotarget.682)
Supplement: Supplementary file 3 [file oncotarget-03-1320-s003.pdf]

Genes affected by mouse mammary tumor virus (MMTV) proviral insertions in mouse mammary tumors are deregulated or mutated in primary human mammary tumors - Callahan et al

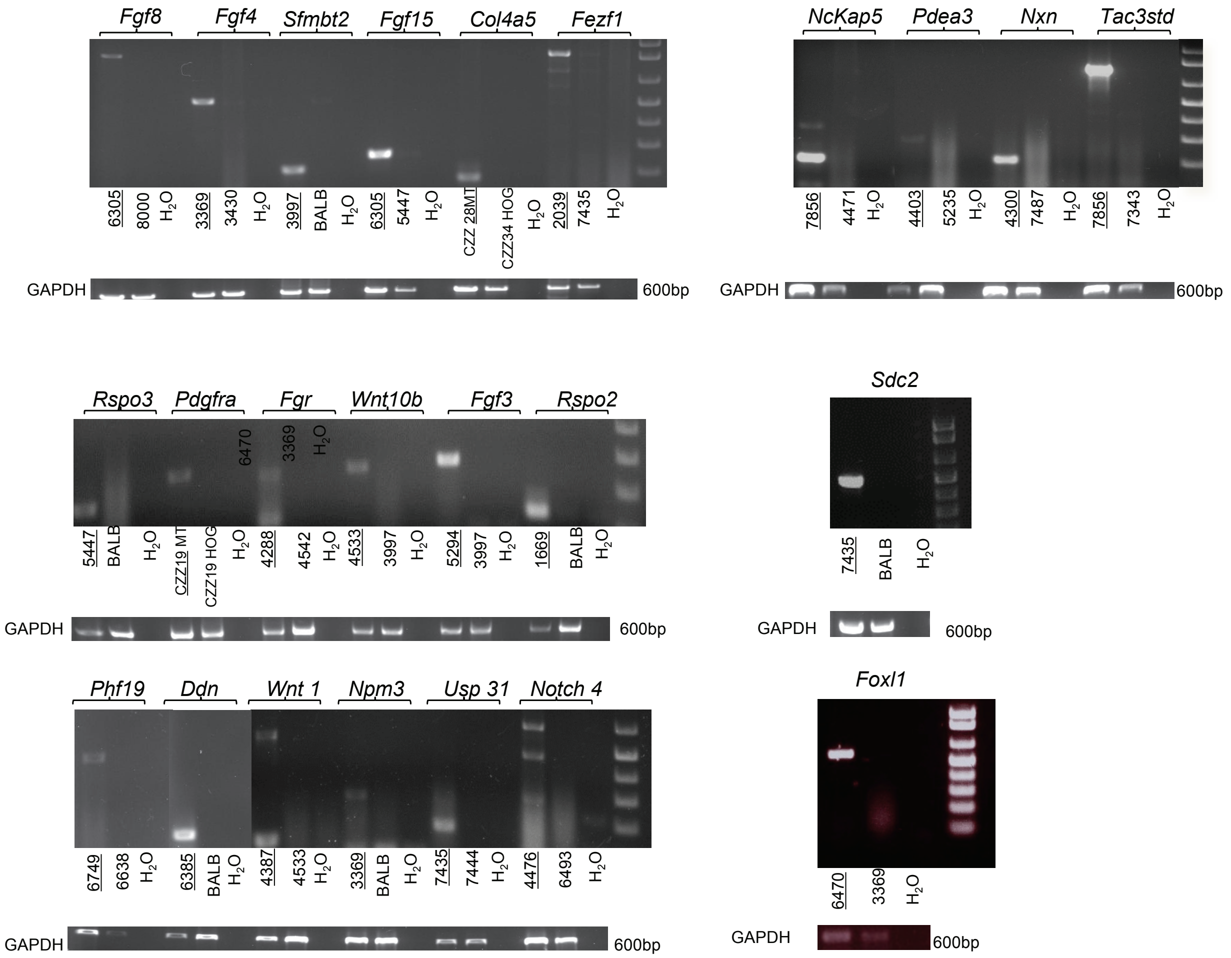

Supplementary Figure 1: RT-PCR verification of microarray analysis of MMTV CIS gene expression in MMTV induced mammary tumors. Agarose gel electrophoresis of the RT-PCR product of tumor RNA was performed for each CIS gene. The product size in base pairs is given in Supplementary Table 1. A representative positive tumor (underlined), a negative tumor or Balb/c mammary gland from day 15 pregnant mouse and water (-) control is shown along with a Gapdh loading control.

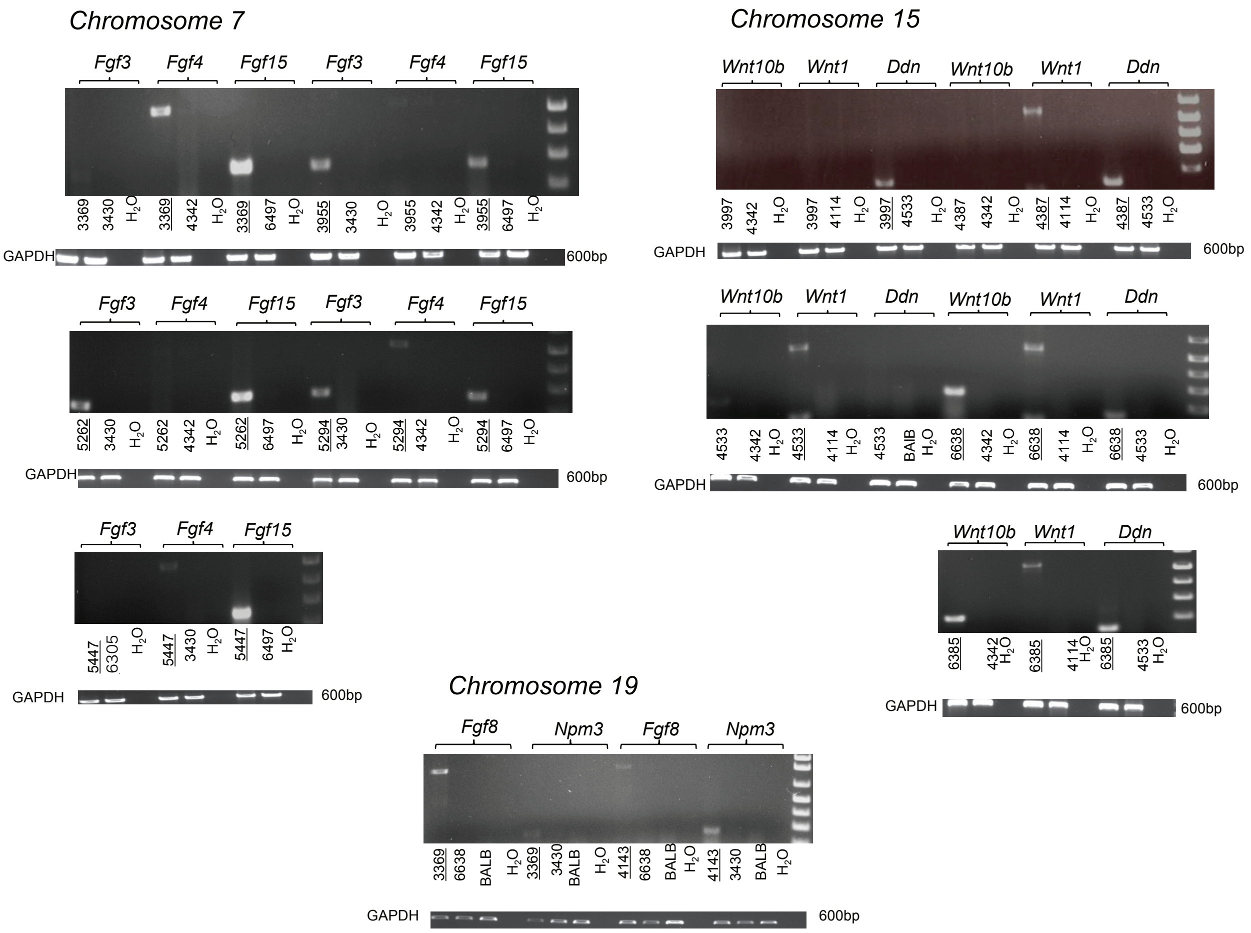

Supplementary Figure 2: RT-PCR analysis of MMTV CIS genes within gene loci on chromosome 7 (Fgf3/Fgf4/Fgf15), chromosome 15 (Wnt1/Wnt10b/Ddn) and chromosome 19 (Fgf8/ Npm3). Agarose gel electrophoresis of the RT-PCR product from a representative tumor RNA was performed for each CIS gene. The product size in base pairs is given in Supplementary Table 1. A positive tumor (underlined), a negative tumor or Balb/c mammary gland from day 15 pregnant mouse and water control is shown along with a Gapdh loading control.

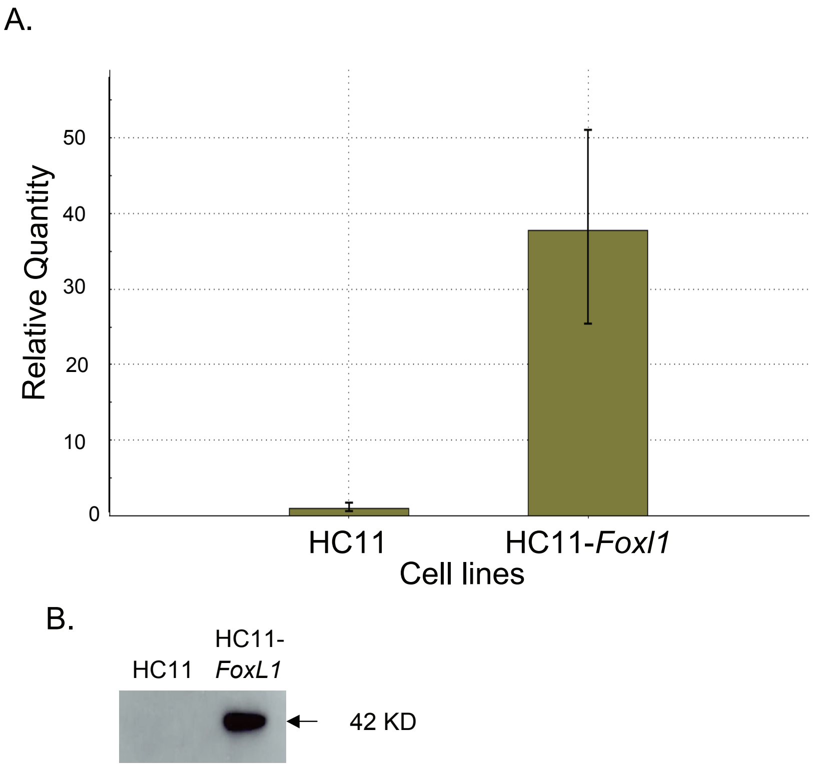

Supplementary Figure 3: Quantitative PCR and Western blot analysis of HC11-Foxl1 for Foxl1 expression. A. Quantitative PCR analysis of HC11 and HC11-Foxl1 total RNA was determined for Foxl1 mRNA relative to Gapdh mRNA. The primers for Foxl1 were Forward 5' GGTCACACTGAACGGCATCTAC 3' and Reverse 5' TCACGAAG CACTCGTTGAGCGA 3'. B. Western blot analysis of HC11 and HC11-Foxl1 protein extract for Foxl1 using antiV5 antibody. The size of the band detected is indicated.

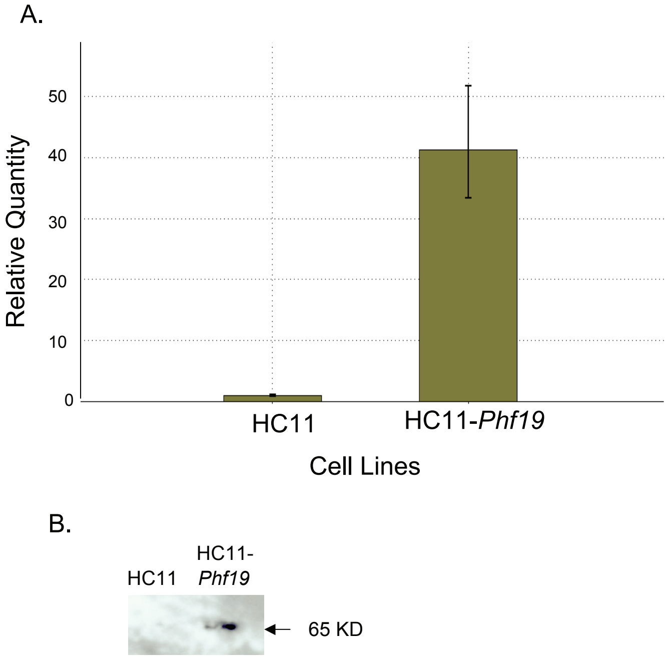

Supplementary Figure 4: Quantitative PCR and Western blot analysis of HC11-Phf19 for Phf19 expression. A. Quantitative PCR analysis of HC11 and HC11-Phf19 total RNA was determined for Phf19 mRNA relative to Gapdh mRNA. The primers for Phf19 were Forward 5' CAGGCAGTGAAAATGGTGCTGTC 3' and Reverse 5' GCACTGTA GCATCCGAAGGTAC 3'. B. Western blot analysis of HC11 and HC11-Phf19 protein extract for Phf19 using antiV5 antibody. The size of the band detected is indicated.

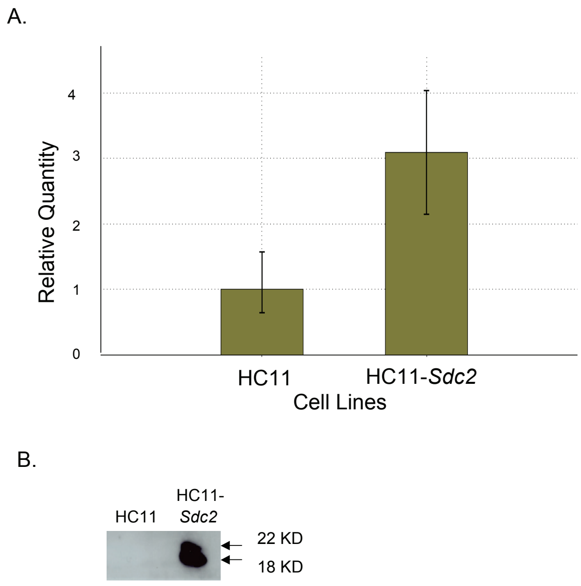

Supplementary Figure 5: Quantitative PCR and Western blot analysis of HC11-Sdc2 for Sdc2 expression. A. Quantitative PCR analysis of HC11 and HC11-Sdc2 total RNA was determined for Sdc2 mRNA relative to Gapdh mRNA. The primers for Sdc2 were Forward 5' GAACAGAGCTGACATCCGATAAG 3' and Reverse 5' GGGATGTT GTCAGAACTGGACTC 3'. B. Western blot analysis of HC11 and HC11-Sdc2 protein extract for Sdc2 using antiV5 antibody. The size of the band detected is indicated.
